# From Basic to Breakthroughs: The Journey of Microfluidic Devices in Hydrogel Droplet Generation

**DOI:** 10.3390/gels11050309

**Published:** 2025-04-22

**Authors:** Gabriela Hinojosa-Ventura, José Manuel Acosta-Cuevas, Carlos Arnulfo Velázquez-Carriles, Diego E. Navarro-López, Miguel Ángel López-Alvarez, Néstor D. Ortega-de la Rosa, Jorge Manuel Silva-Jara

**Affiliations:** 1 Departamento de Farmacobiología, Centro Universitario de Ciencias Exactas e Ingenierías, Universidad de Guadalajara, Guadalajara 44430, Mexico; 2 Departamento de Innovación Tecnológica, Centro Universitario de Tlajomulco, Universidad de Guadalajara, Tlajomulco de Zúñiga 45641, Mexico; 3 Departamento de Ingeniería Química, Centro Universitario de Ciencias Exactas e Ingenierías, Universidad de Guadalajara, Guadalajara 44430, Mexico; 4 Departamento de Ingeniería Biológica, Sintética y de Materiales, Centro Universitairo de Tlajomulco, Universidad de Guadalajara, Tlajomulco de Zúñiga 45641, Mexico; 5 Escuela de Ingeniería y Ciencias, Tecnológico de Monterrey, Zapopan 45138, Mexico; 6 Departamento de Ingeniería Mecánica, Universidad de Guadalajara, Guadalajara 44430, Mexico

**Keywords:** hydrogels, microhydrogels, microfluidic devices, microfluidic methods, materials for microfluidic devices, biomedical applications

## Abstract

Hydrogel particles are essential in biological applications because of their distinctive capacity to retain water and encapsulate active molecules within their three-dimensional structure. Typical particle sizes range from nanometers (10–500 nm) to micrometers (1–500 µm), depending on the specific application and method of preparation. These characteristics render them optimal carriers for the administration of active compounds, facilitating the regulated and prolonged release of pharmaceuticals, including anticancer agents, antibiotics, and therapeutic proteins. Hydrogel particles can exhibit various morphologies, including spherical, rod-shaped, disk-shaped, and core–shell structures. Each shape offers distinct advantages, such as improved circulation time, targeted drug delivery, or enhanced cellular uptake. Additionally, hydrogel particles can be engineered to respond to various stimuli, such as temperature, pH, light, magnetic fields, and biochemical signals. Furthermore, their biocompatibility and capacity to acclimate to many biological conditions make them appropriate for sophisticated applications, including gene treatments, tissue regeneration, and cell therapies. Microfluidics has transformed the creation of hydrogel particles, providing precise control over their dimensions, morphology, and stability. This technique facilitates reproducible and highly efficient production, reducing reagent waste and optimizing drug encapsulation. The integration of microfluidics with hydrogels provides opportunities for the advancement of creative and effective solutions in contemporary medicine.

## 1. Introduction

In recent years, nanomedicines have significantly changed the fields of science and medicine. They are being developed to treat numerous chronic diseases such as cancer, diabetes, Alzheimer’s, and multiple sclerosis; infectious diseases; autoimmune diseases; and metabolic disorders [1,2,3]. Nanofomulations were essential for the creation and stability of mRNA vaccines [4,5] and in immunotherapy and regenerative medicine [1,2,3]. Moreover, high-tech imaging methods and high-sensitivity biosensors have been used for early detection of diseases like cancer [6,7]. Another use of hydrogel-based nanofunctionalized materials is in nutrition, where they greatly improve food safety, preservation, and the availability of nutrients and beneficial compounds. Biocompatible and biodegradable hydrogels can be used as safe vehicles to encapsulate and protect active compounds in functional foods, supplements, and probiotics. Moreover, their ability to respond to specific environmental stimuli allows for the controlled release of active ingredients, thereby improving the quality, shelf life, and nutritional efficacy of foods [8,9,10].

Crosslinked polymers that contain water form hydrogels, which can hold drugs, biomolecules, or living organisms [11,12,13]. Some hydrogels, such as chitosan [14], hyaluronic acid [15], alginate [16], and collagen [17], have proven to be safe for use in biological tissues because they are biocompatible and biodegradable [1,11,18,19].

The controlled and targeted release of drugs has been effectively achieved. They are considered natural self-adhesives [20,21] and have shown resistance to protein adhesion [22,23]. On the other hand, the bioavailability of these medications allows for lower doses, which mitigates toxicity and some of the problems associated with treatments [24,25,26]. They are distinguished by their reactions to external stimuli such as pH, temperature, or light [12,27,28,29,30,31]. They also prevent the immune system’s reaction to an external substance within the body [32,33].

**Figure 1:**
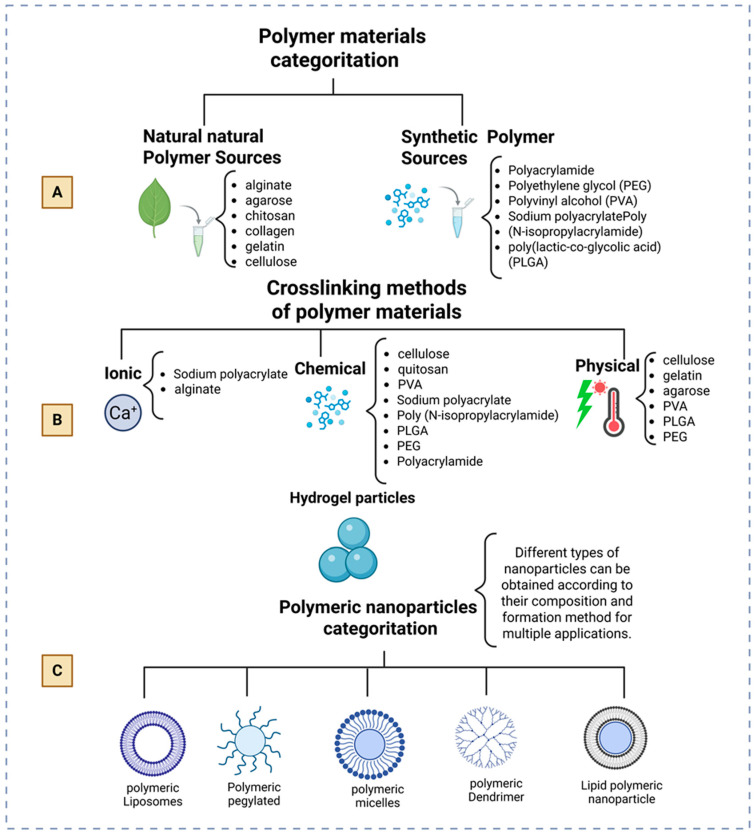
(**A**) Classification of polymer materials; (**B**) Crosslinking processes of polymer materials; (**C**) Common nanoformulations bases on polymer materials applied in medicine.

Figure 1 illustrates the fabrication of hydrogel particles, starting with the materials utilized, following through the solidification techniques, and concluding with the diverse categories of polymeric nanoparticles. Figure 1A illustrates the most basic method for classifying polymeric materials into natural and synthetic polymers. It delineates the fundamental classification of polymeric materials into natural and synthetic polymers. Examples of each class are also presented according to their chemical properties. Natural polymers originate from biological sources, including plants and animals, and are often biodegradable and environmentally friendly. However, synthetic polymers are generally stronger and diverse in their characteristics. Every polymer type possesses distinct advantages and specific uses across many industries. Natural polymers, also known as biopolymers, include chitosan, hyaluronic acid, dextran, k-carrageenan, guar gum, chondroitin sulfate (derived from polysaccharides), gelatin, fibroin, keratin (derived from proteins), among others, as specified in references [1,12,13,20].

The synthetic hydrogels include Poly(ethylene) Glycol Diacrylate (PEGDA), poly(methyl methacrylate-co-acrylic acid) (P(MMA-co-AA)), poly(hydroxyethyl methacrylate) (PHEMA), poly(vinyl alcohol) (PVA), methacrylic acid (MAA), N-vinyl pyrrolidone (NVP), PEG monomethyl ether monomethacrylate (PEGMMA), and poly(lactic-co-glycolic acid) (PLGA) [1,12,22,28,34].

Figure 1B illustrates various hydrogel crosslinking processes, along with examples of each type. Various crosslinking forms are utilized based on the particular materials and their biological or pharmacological purposes. Physical crosslinking employs non-covalent interactions, including hydrogen bonds, hydrophobic contacts, ionic connections, and techniques such as freezing and thawing cycles. Crosslinked hydrogels frequently establish reversible and stimulus-responsive networks, enabling them to adapt dynamically to environmental fluctuations, including changes in temperature or pH [11].

Chemical crosslinking, conversely, forms stable covalent connections by utilizing crosslinking agents or by harnessing reactive functional groups present in the polymer chains. This method provides enhanced mechanical and chemical stability, which is advantageous for prolonged and regulated drug delivery applications and structural reinforcement in tissue engineering [11,35]. Alternative approaches include photopolymerization, a process that uses ultraviolet or visible light in conjunction with specific chemicals known as photoinitiators to rapidly and accurately form a network of interconnected molecules. An instance of photopolymerization occurs with poly (ethylene glycol) diacrylate (PEGDA) upon exposure to ultraviolet light [36]. Conversely, enzymatic crosslinking utilizes enzyme-catalyzed processes to facilitate the synthesis of hydrogels under mild and biocompatible conditions [37]. Ultimately, radiation-induced crosslinking entails the use of gamma radiation or electron beam exposure to create covalent connections independently of supplementary crosslinkers or chemical initiators [38]. The diverse crosslinking methods, among others [39], offer significant flexibility to accurately customize the hydrogel’s characteristics, fulfilling the specific demands of a wide range of biological and pharmaceutical applications.

Collectively, these diverse crosslinking techniques, along with additional ways [39], offer extensive flexibility to meticulously customize the hydrogel’s characteristics, fulfilling the unique demands of a wide range of biological and pharmaceutical applications.

Figure 1 illustrates the fabrication of hydrogel particles, first with the materials utilized, followed by the techniques employed to solidify the particles, and concluding with the diverse categories of polymeric nanoparticles. Figure 1A illustrates the most fundamental method of classifying polymeric materials into natural and synthetic polymers.

Figure 1A delineates the fundamental classification of polymeric materials into natural and manmade polymers. Examples of each category are shown according to their chemical composition. Natural polymers originate from biological sources, including flora and fauna; they are typically biodegradable and ecologically sustainable, whereas synthetic polymers are generally more resilient and adaptable in their characteristics. Every polymer type possesses distinct advantages and specific uses across many industries. Natural polymers, also known as biopolymers, encompass chitosan, hyaluronic acid, dextran, k-carrageenan, guar gum, chondroitin sulfate (derived from polysaccharides), gelatin, fibroin, keratin (derived from proteins), among others, as specified in references [1,12,13,20].

Figure 1C illustrates a range of nanoformulations using polymeric materials utilized in contemporary medicine. Researchers have examined hydrogels individually or in combination for their use as drug delivery systems. Lucía Martín-Banderas investigated PLGA for gemcitabine entrapment in 2013, while Carla B. Roces employed PLGA as vaccine adjuvants in 2020 [40,41]. Polymeric materials integrated with other molecules yield a diverse array of nanoformulations. Polymeric liposomes, which include copolymers along with other ingredients like lipids and cholesterol, improve how well drugs are delivered and help target specific cells or areas. In 2014, Liu et al. [42] created liposomes made from dextran that contained paclitaxel and methotrexate to deliver multiple drugs in a controlled way during chemotherapy. Various writers have employed liposomes for the release of RNA [43,44].

Pegylated particles consist of polymers conjugated with polyethylene glycol (PEG) chains, enhancing the solubility, stability, and targeting efficacy of pharmaceuticals; they are utilized for the sustained delivery of medications to specified cells [45,46,47]. Lipids and polymers combine to form lipid polymer nanoparticles, which serve as a versatile drug delivery method. These nanoparticles have shown promise in enhancing drug stability and bioavailability for targeting specific tissues or cells within the body [48,49,50]. Micelles and polymeric dendrimers are different structures that have been carefully combined with hydrogels for medical uses to help carry medicines inside them, including many drugs [1,21,31]. As research progresses, it becomes clearer that these materials can effectively address complex medical problems with better precision in areas like targeted drug delivery, regenerative medicine, diagnostics, and vaccines. As research progresses, it becomes clearer that these materials can effectively address complex medical problems with better precision in areas like targeted drug delivery, regenerative medicine, diagnostics, and vaccines.

## 2. Biomedical Applications of Polymeric Nanoparticles

**Figure 2:**
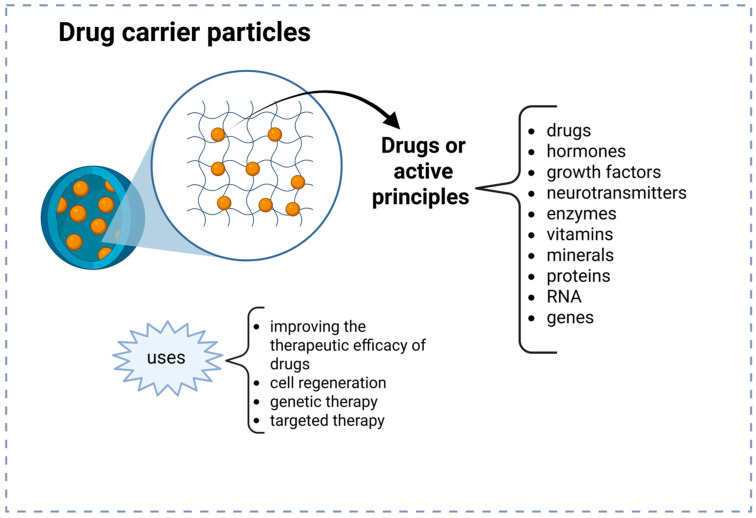
Biomedical application of hydrogel nanoparticles for drug delivery. Yellow circles represent drugs or active principles encapsulated.

Figure 2 delineates the biomedical applications of polymeric nanoparticles classified into two categories. Particles encapsulated with drugs and living cells. Figure 2 depicts an image in which the hydrogel particle, referred to as a carrier or vehicle in medicine, encapsulates medications or active principles. The literature indicates that many substances, including hormones, growth factors, enzymes, vitamins, proteins, and RNA, have been encapsulated into a polymeric nanoparticle for the treatment of diseases such as cancer, diabetes, and cardiovascular ailments. These particles present a promising approach for targeted drug delivery, enhancing the therapeutic efficacy of pharmaceuticals, cellular regeneration, gene therapy, targeted therapy, and personalized medicine. To further enhance hydrogel functionality, active molecules such as pharmaceuticals, hormones, growth factors, enzymes, vitamins, therapeutic proteins, and nucleic acids (RNA/DNA) are routinely integrated within these polymer matrices. These encapsulated bioactive molecules allow hydrogels to serve multiple therapeutic purposes, ranging from controlled drug delivery to regenerative medicine applications [1,12,19,20,35].

**Figure 3:**
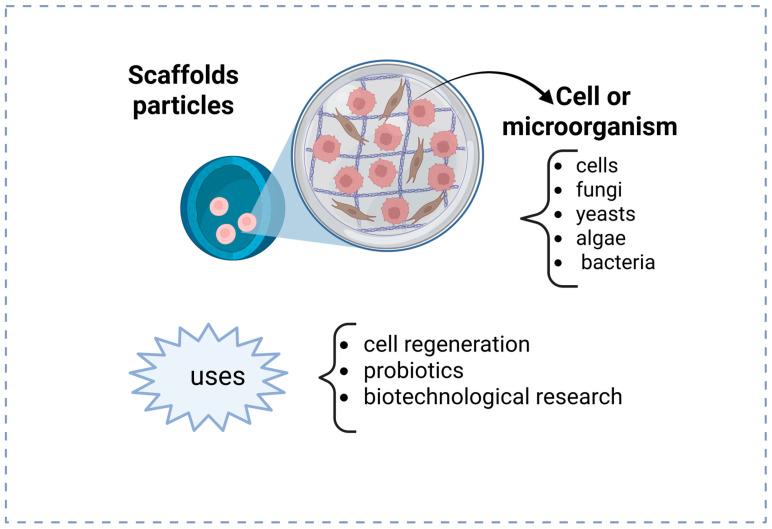
Biomedical application of hydrogel nanoparticles as scaffolds. Pink and brown digures represent cell or microorganisms encapsulated.

Particles of live cell-encapsulated in hydrogel, depicted in Figure 3, are predominantly utilized as scaffolds in in situ-forming research. Potential for regenerative medicine has been demonstrated, particularly in tissue, cartilage, and bone engineering applications, by creating a microenvironment that supports cell development and function. Another application is in biotechnological engineering, cultivating microorganisms capable of generating beneficial metabolites. Additionally, probiotic bacteria have been encapsulated for enhancement in promoting gut health and supporting the immune system.

Matrices or cellular scaffolds have been extensively utilized to encapsulate cells for tissue regeneration purposes, particularly in applications involving cartilage and bone, and are broadly employed in in situ-forming research [12,35,51,52]. These hydrogel scaffolds typically feature internal architectures characterized by interconnected voids, hollows, or pores. The size and distribution of these pores depend directly on the desired encapsulation objectives. For instance, when aiming to immobilize microorganisms or cells, pore sizes must be smaller than the encapsulated entities to prevent their undesired diffusion through the scaffold structure.

Encapsulation within polymeric hydrogel nanoparticles commonly utilizes methods such as ionic gelation, where ionic interactions form crosslinked polymer networks encapsulating cells or biomolecules gently and efficiently. Another prevalent method is microfluidic encapsulation, which allows precise control over particle size and uniformity, thus enhancing encapsulation efficiency and cell viability. Recent studies have demonstrated successful encapsulation of mammalian cells and microorganisms, such as Escherichia coli, using microfluidic-generated hydrogel particles [53]. The controlled release from hydrogel nanoparticles relies primarily on diffusion when encapsulated substances slowly migrate out through the hydrogel network’s micropores; polymer matrix degradation polymers suffer a gradual degradation under physiological conditions, liberating the encapsulated agents at predictable rates or responsiveness The controlled release from hydrogel nanoparticles relies primarily on diffusion when encapsulated substances slowly migrate out through the hydrogel network’s micropores; polymers in the matrix gradually degrade under physiological conditions, liberating the encapsulated agents at predictable rates or in response to environmental stimuli such as pH, temperature, enzymes, or external triggers like light.

Hydrogel nanoparticles are also effectively employed to isolate and encapsulate microorganisms, including beneficial probiotic bacteria and pathogens. Studies have reported the successful encapsulation and controlled release of probiotics like *Bifidobacterium bifidum* and *Lactobacillus rhamnosus* for enhanced gut colonization and immune modulation [54]. Conversely, encapsulation has been explored to isolate harmful pathogens such as *Escherichia coli* strains to safely evaluate antimicrobial treatments [12,35].

## 3. Hydrogel Particles Morphology

Three-dimensional polymeric networks constituted of natural, synthetic, or hybrid hydrogels are distinguished by their exceptional ability to retain water [22,35]. Crosslinking is essential for hydrogel formation and is possible by chemical (covalent bonds) or physical (non-covalent interactions like Van der Waals forces) methods [35]. The nature of crosslinking imparts structural stability and swelling capability to hydrogels. The crosslinking density directly influences characteristics, including elasticity, mechanical strength, and pore size. Common crosslinkers include poly (ethylene glycol) (PEG), poly (hydroxyethyl methacrylate) (PHEMA), and poly (vinyl alcohol) (PVA), which are photopolymerizable hydrogels [13].

**Figure 4:**
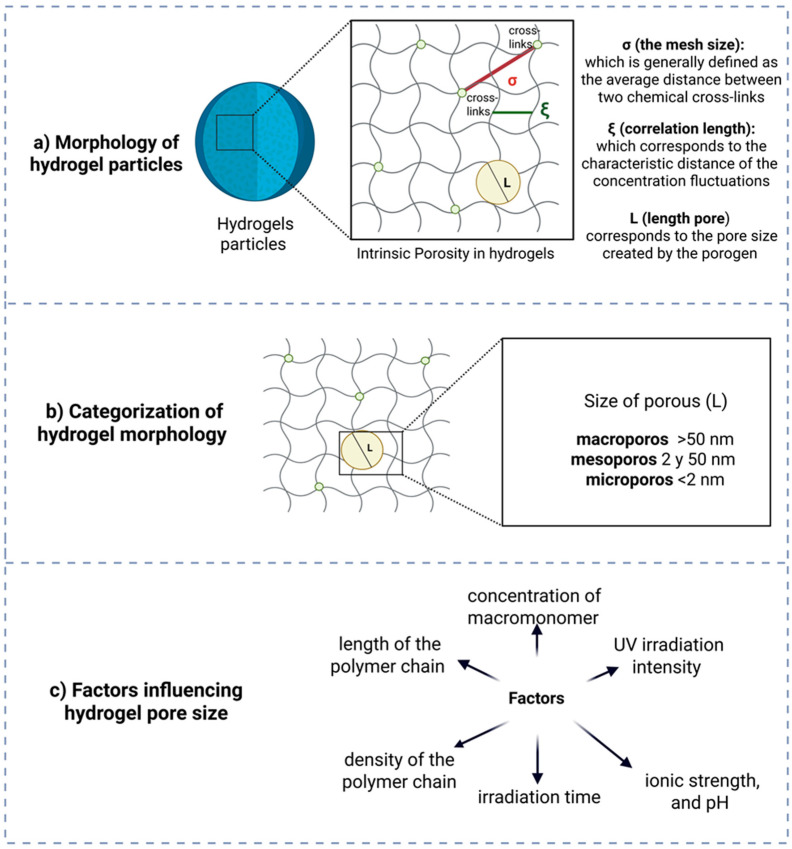
(**a**) Morphology of hydrogel particles. (**b**) Categorization of hydrogel morphology according to pore size. (**c**) Factors influencing hydrogel pore size.

There are spaces between the polymer filaments in the hydrogel network. This is called intrinsic porosity, and it is shown by the mesh size [13]. The size of the pores and their distribution in hydrogels are important design parameters for the development of biomaterials as they directly affect their transport or diffusion properties. The swelling capacity of the material influences the hydrogel’s ability to facilitate the flow of molecules, including nutrients or medicines, and its structural functions in tissue engineering (Figure 4a) [28,29].

Hydrogels can be categorized based on pore size into three primary classifications. Microporous materials possess pores smaller than 2 nm, making them suitable for the exclusion of diminutive molecules. Mesoporous materials have pores ranging from 2 to 50 nm and are frequently employed for controlled medication delivery and biomolecule encapsulation. Macroporous materials exhibit pores larger than 50 nm, making them appropriate for applications like tissue engineering, as they facilitate the movement of cells and nutrients (Figure 4b) [13].

The formulation of hydrogels can modify physical and mechanical properties, including swelling capacity, soluble fraction, compression, crosslinking density, and pore size, to obtain the desired release profiles (Figure 4c) [12,13,18,19].

The concentration of monomer utilized in hydrogel synthesis directly influences the crosslink density of the polymer network. As the monomer concentration increases, the network becomes progressively dense, leading to lower pore size and a reduced swelling capacity [22,30,36].

Pore size is affected by multiple factors. Elevated crosslink density diminishes pore size by augmenting the junction points within the network. The polymer’s composition, encompassing its flexibility and swelling capacity, directly influences the pore structure. The synthesis parameters, including temperature, pH, and reactant concentration, are also significant factors [28]. The degree of hydrogel swelling in a particular medium could affect the size of the pores. The inclusion of additives like surfactants or additives can modify the structure and overall dimensions of the pores [13].

UV radiation is commonly employed in hydrogels to increase crosslink density, thereby reducing pore size and swelling capacity due to enhanced polymer cohesion [13,36]. Factors such as ionic strength, salt concentration, and environmental pH significantly influence the polymer network’s swelling behavior by altering its ionic charge and its affinity for water molecules. For instance, variations in pH can protonate or deprotonate functional groups within the polymer chains, modifying electrostatic repulsion and consequently adjusting the swelling capacity [55,56]. Typically, an optimal pH induces sufficient electrostatic repulsion among polymer chains, resulting in increased swelling due to network expansion. Additionally, exposure duration and intensity of UV radiation directly impact crosslinking degree, further controlling the internal porosity and swelling characteristics of hydrogels [57]. To precisely determine these effects, experimental validations using methods such as swelling kinetics studies, scanning electron microscopy (SEM) analysis of pore structures, and rheological measurements of hydrogel elasticity are usually employed [55,57].

The density of a polymer chain affects its crosslinking structure, reducing pore size and limiting water absorption. Longer chains can establish networks with greater flexibility and extensive pores, facilitating wider water absorption. Both factors contribute to the overall structure of the polymer chain [27,31,36].

## 4. Microfluidics and Its Biomedical Applications

Microfluidics is the field that examines the behavior of fluids and the creation of devices with micro- and nanometer-scale channels [58,59,60]. The purpose of microfluidics development was to miniaturize laboratory equipment and enhance the effectiveness of utilizing mixes, chemicals, and samples [61,62]. Microfluidics, which is defined as the study, design and manipulation of fluids in micrometer-scale and nanometer-scale channels where surface forces and diffusion dominate, enabling the creation of integrated, miniaturized laboratory systems for efficient analysis and processing [63,64], is a field relevant to numerous domains, including biomedicine, analytical chemistry, materials science, environmental monitoring, nanotechnology, biotechnology, biochemistry, physics, and engineering [65].

Droplet generation occurs through two primary mechanisms: (1) active methods, necessitating external forces, and (2) passive methods, reliant on channel geometry and phase arrangement [66]. The small sample size needed and the capacity for point-of-care testing (POCT) make these devices ideal for rapid and accessible diagnostics. Microfluidic devices in diagnostics facilitate rapid and accurate analyses of samples, including blood, urine, and other bodily fluids, to detect infectious and chronic diseases such as HIV, hepatitis, malaria, and COVID-19 [62,67].

Microfluidics is an emerging field of science that originated in the mid-19th century, inspired by advancements in electronics that resulted in the development of the first transistors and semiconductors, permitting the precise manipulation and fabrication of minuscule structures [68]. Initially, the devices were mostly constructed from silicon and glass, which enabled accurate manipulation of the microchannels [69,70,71,72]. These are appropriate for applications necessitating enhanced chemical and thermal resistance [69,73,74,75,76].

Polydimethylsiloxane (PDMS) gained popularity in the 1990s due to its reasonable price, flexibility, and simplicity of molding as an elastomer. This facilitated the design and fabrication of microfluidic devices [60,72,77]. Moreover, PDMS is a transparent material applicable in many biomedical diagnostics, biological science research, and real-time studies [74,75,78]. PDMS is among the most prevalent materials utilized in biological applications because of these factors. It is also accountable for the development of lab-on-a-chip (LOC) devices, enabling comprehensive laboratory tests to be conducted on a compact, portable device [72,78,79,80].

**Figure 5:**
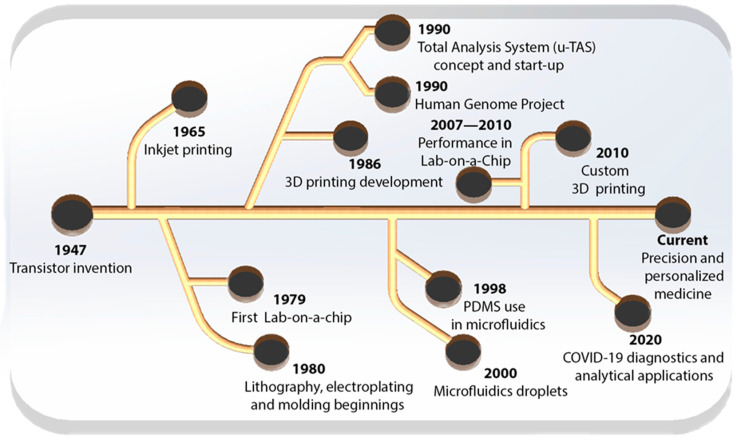
Chronology of microfluidics development [81,82,83,84,85,86,87,88,89].

Numerous polymeric materials are widely utilized in the fabrication of microfluidic devices, including polymethyl methacrylate (PMMA), photo-curable resins, polyimide, polyurethane, polyester, and polypropylene, among others. The infrastructure and application of these devices dictate the utilization of these materials. Paper is the base material utilized in the fabrication of microfluidic devices (μPADs), demonstrating superiority in diagnostic applications owing to its low price, manufacturing simplicity, and disposability. Figure 5 illustrates notable developments in the history of microfluidics.

## 5. Geometric Configurations of Microfluidic Devices

**Figure 6:**
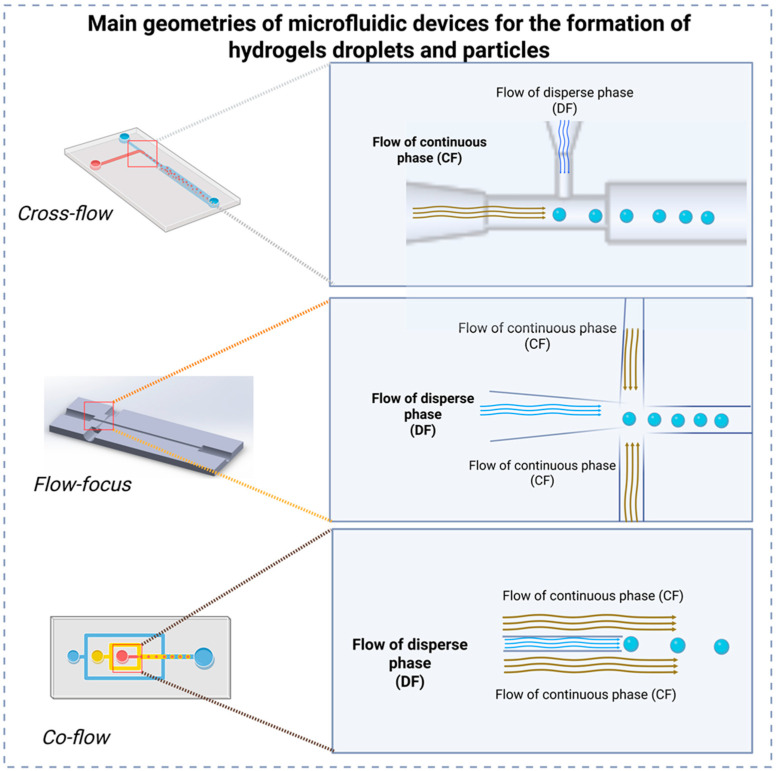
Main geometries of microfluidic devices for the formation of hydrogels. The left side illustrates the diapositives used for each geometry, where different colors can be continuous or flow phases. On the right side, the flow and formation of droplets (blue circles) is depicted.

In passive methods, the most frequently employed microfluidic device configurations are crossflow, flow-focusing, and co-flow [66]. Each configuration provides distinct advantages regarding the precise control of droplet formation, emulsification, and double emulsion generation (Figure 6).

Among these, flow-focusing devices stand out due to their ability to consistently produce highly monodisperse and spherical droplets at elevated production rates, making them particularly suitable for encapsulation processes and controlled emulsification [90,91,92]. The flow-focusing geometry operates by symmetrically introducing the dispersed phase through a central channel, surrounded by the continuous phase entering laterally. This configuration generates a uniform shear force around the dispersed fluid, facilitating stable and uniform droplet breakup. Such precise control makes flow-focusing ideal for drug delivery systems, rapid emulsification processes, and microreactors requiring exact mixing conditions [52,93].

The crossflow configuration relies on the shear force exerted by the continuous phase, perpendicular to the dispersed phase, combined with interfacial tension to regulate droplet formation. This design efficiently produces droplets with controlled and predictable sizes, suitable for creating monodisperse emulsions and single-droplet microreactors commonly used in chemical and biological analyses [94,95,96]. Crossflow geometry is advantageous when rapid and reliable droplet production is essential, owing to its straightforward design and ease of operation.

Finally, the co-flow configuration employs coaxial channels wherein the dispersed phase flows through an inner channel surrounded concentrically by the continuous phase. This arrangement ensures reduced turbulence and gentle shear forces, which minimize potential damage to sensitive encapsulated materials such as cells, biomolecules, or delicate active substances [34,52]. Co-flow is particularly effective for generating stable droplets, single emulsions, or sophisticated double emulsions—systems characterized by droplets encapsulated within droplets—which are extensively used in microencapsulation applications requiring high precision and uniformity [52].

In all these geometries, the careful selection of fluid properties (such as viscosity and interfacial tension) and the strategic use of surfactants are critical to achieving optimal droplet stability, preventing undesired coalescence, and ensuring consistent droplet or particle formation [93,97].

## 6. Materials and Methods Used for Device Fabrication

The selection of materials for microfluidic devices is predominantly dictated by their intended purpose. The materials employed in the construction of microfluidic devices are essential, necessitating substances with physical, chemical, and mechanical properties that satisfy the requirements of micrometric and nanometric scale systems, including biocompatibility, optical transparency, suitable mechanical characteristics, and fabrication feasibility. The material employed in the construction of microfluidic devices dictates the functional characteristics and the relevant manufacturing methods [98]. Microfluidics materials are classified into three primary categories: inorganic substances, polymers, and paper. Their selection is contingent upon physicochemical features, biocompatibility, fabrication ease, and price [99].

Silicon is a crystalline semiconductor extensively utilized in microtechnology because of its compatibility with conventional manufacturing techniques, including optical lithography and reactive etching. This material displays a crystalline structure that facilitates the creation of channels and microstructures with straight and precise walls. Furthermore, its elevated thermal conductivity is optimal for dissipating heat in operations that produce substantial thermal fluctuations [100]. Silicon’s surface can undergo chemical modification via coatings to enhance its suitability for various applications, including improved liquid wetting and inhibition of biomolecule adsorption; nonetheless, its application is limited to devices necessitating direct optical observation [101,102,103].

Glass is an amorphous substance valued for its resistance to chemical corrosion and its excellent transparency in both the visible and ultraviolet spectra. Wet or dry chemical etching methods facilitate the creation of microfluidic channels with rounded profiles, ideal for consistent laminar flows. The chemical composition can differ based on the glass type, ranging from borosilicate glass (resistant to heat and chemicals) to quartz glass (characterized by high purity and transparency) [101,104]. Glass facilitates the incorporation of electrodes and additional functional components via techniques such as metal deposition or dielectric coatings, hence enhancing its applicability in microelectrochemical systems [105,106,107].

The ceramics employed in microfluidics, particularly those utilizing LTCC (Low Temperature Co-fired Ceramics) technology, are polycrystalline materials characterized by superior mechanical and thermal durability. In the production process, ceramics are sintered at low temperatures, facilitating the incorporation of conductive materials like platinum or copper into the device structure [33,101,108]. The channels and chambers are constructed using stamping or cutting methods prior to the final firing, facilitating the production of intricate three-dimensional shapes. Ceramics are suitable for applications necessitating resistance to aggressive solvents and elevated pressures [100,109].

Thermosetting polymers, including PDMS (polydimethylsiloxane) and photosensitive epoxy resins, are crosslinked substances that do not soften with heating. PDMS is a transparent elastomer characterized by significant elasticity and facile mold replication, predominantly utilized in soft lithography. A photosensitive epoxy resin facilitates the fabrication of stiff microstructures with high resolution by photopolymerization methods utilizing suitable equipment [101,105,110].

Thermoplastics, including PMMA (polymethyl methacrylate), PC (polycarbonate), and PS (polystyrene), are linear or marginally branched polymeric substances that may be remolded multiple times with the application of heat. These polymers possess distinct glass transition temperatures (Tg), beyond which they attain malleability, facilitating production techniques such as injection molding or hot stamping [102,111,112]. Thermoplastics integrate stiff and flexible segments within their molecular architecture, imparting elasticity and heat processability. These materials exhibit significant versatility, with mechanical qualities modifiable via alterations in their composition [100,113,114].

Hydrogels are hydrophilic substances composed of three-dimensional polymer networks capable of absorbing substantial quantities of water without disintegration. Their porous architecture enables the regulated diffusion of small chemicals and promotes the integration of living cells in biological applications [100,115]. Hydrogels can be manufactured via chemical or physical crosslinking reactions, allowing for the modification of their mechanical characteristics, swelling capacity, and permeability by altering their composition or fabrication circumstances. Typical instances encompass natural polymers (alginate, collagen) and synthetic polymers (polyacrylamide, sodium polyacrylate, PNIPA, PEG) [105,116,117].

Biodegradable materials, like PGA (polyglycolic acid) and PCL (polycaprolactone), are polymers engineered to decompose under biological conditions. Their structure comprises chemical bonds that are vulnerable to hydrolysis or enzymatic activity. These materials can be produced by extrusion processes, 3D printing, or solvent casting, facilitating intricate structures for temporary applications. They provide biodegradable microstructured platforms for drug administration and tissue scaffolding [100,101,118].

The paper utilized in microfluidics is a porous substance composed of cellulose fibers. Its configuration facilitates the passive movement of liquids by capillarity, obviating the necessity for external pumps. Microfluidic patterns are established through the application of hydrophobic inks or chemical treatments, delineating channels and reaction zones [101,119]. The paper’s thickness, density, and surface treatment can be modified to enhance liquid retention, flow rate, and sensitivity in diagnostic assays [105,120]. Table 1 delineates many research investigations utilizing microfluidic devices to produce particles having biomedical applications.

## 7. Droplet Formation Through Microfluidics

Microfluidics is utilized for the manipulation of materials as monodisperse droplets at micro- and nanometric scales, referred to as droplet-based microfluidics [93]. The production of droplets commonly involves at least two immiscible liquids, designated as the continuous phase (CF) and the dispersion phase (DF). The continuous phase acts as the medium for droplet formation, whereas the dispersed phase comprises the droplets themselves [58].

Emulsions are categorized based on the interaction of these phases as water in oil (W/O), oil in water (O/W), water in oil in water (W/O/W), and oil in water in oil (O/W/O). Understanding the characteristics and the chemical and physical properties of the materials used to create emulsions is essential, as the formation of emulsions necessitates a fluid of differing nature to ensure their immiscibility [66].

Microfluidic methods significantly surpass traditional approaches due to their capacity for precise and reproducible control over droplet or particle formation [42,117,139]. Traditional methods, such as emulsification through mechanical stirring or sonication, typically produce particles with broad size distributions, inconsistent morphologies, and limited reproducibility due to heterogeneous shear forces and uncontrolled fluid dynamics [60,61]. In contrast, microfluidic devices precisely manipulate fluid flows at the microscale, ensuring consistent shear and interfacial forces. This precise regulation results in uniform droplets or particles with highly controllable dimensions and shapes [13,141].

Moreover, microfluidic techniques allow meticulous tuning of flow rates and channel geometries, enabling reproducible and reliable particle generation even at high production frequencies. The droplets generated can be further functionalized to respond accurately and predictably to various triggers, including chemical stimuli, temperature fluctuations, or exposure to UV radiation, to initiate controlled polymerization or gelation processes [13,141]. Consequently, microfluidic methods ensure enhanced batch-to-batch reproducibility, higher efficiency in reagent utilization, reduced waste, and superior control over particle properties compared with conventional bulk preparation methods [123,128].

**Figure 7:**
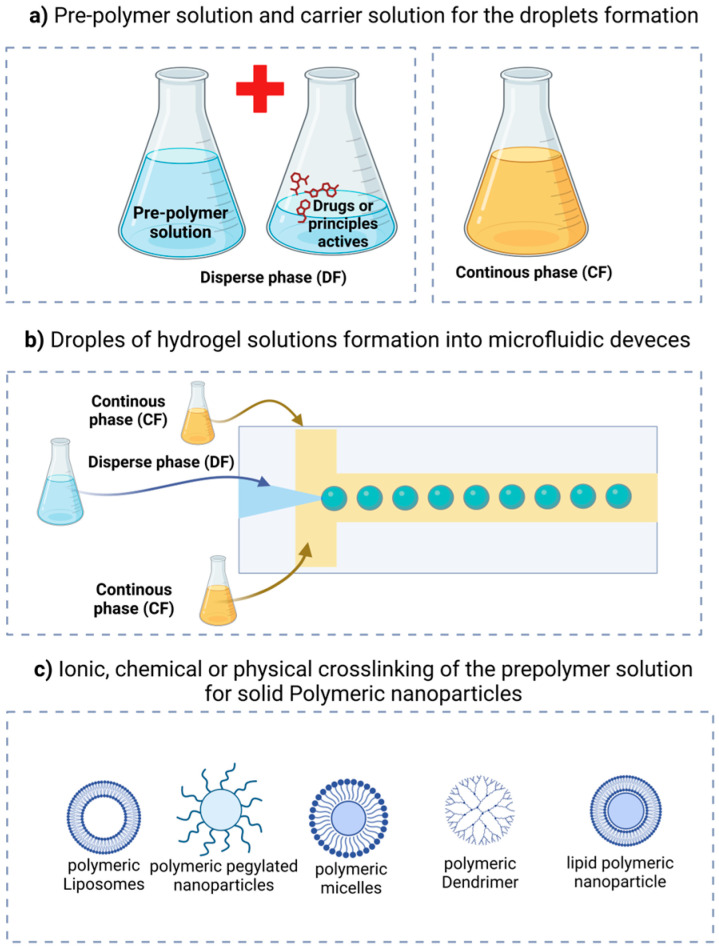
Hydrogel particle formation within a microfluidic device: (**a**) dispersed and continuous phases; (**b**) formation of pre-polymeric droplets; and (**c**) possible polymeric nanoparticles.

Figure 7 illustrates the continuous phase, which encompasses and transports the droplets (often oil in various systems), alongside the dispersed phase, consisting of droplets from the pre-polymeric hydrogel solution. Based on the chemical composition of the pre-polymer in the dispersed phase, a chemical or physical stimulus (such as temperature variations or ultraviolet radiation) is necessary to initiate the polymerization or crosslinking of the hydrogel, converting it into solid or semi-solid particles [35].

**Figure 8:**
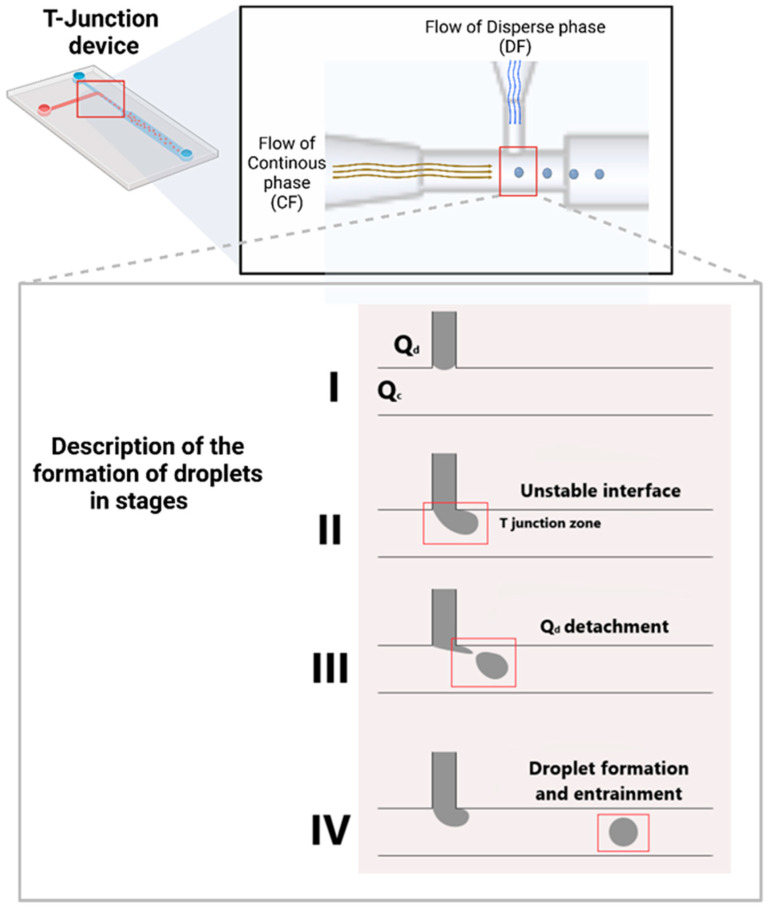
Development droplets within a T-junction microfluidic device. The red square follows the formation of the droplet in the device.

Figure 8 illustrates the development of a droplet within a microfluidic device. In the DF channel, the entrance of the dispersion phase is located, while the CF channel facilitates the flow of the continuous phase, which shears and transports the dispersed phase droplets toward the exit channel. Zone I illustrates the entry point of the DF fluid, while Zone II is designated as Zone T based on the channel shape. The droplet creation is visible in this zone. In Zone III, the droplet separates due to the pull exerted by the CF fluid, and ultimately, in Zone IV, the droplet traverses the output channel without intermingling the two fluids. Fluids with polymer precursors or hydrogel substances are put into the microfluidic device during this step [60].

## 8. Nanomedicines Based on Hydrogels Formed Through Microfluidics

Electrohydrodynamic spraying, 3D bioprinting, microemulsion, dispersion/precipitation, polymerization, physical self-assembly, and supramolecular host–guest interactions are some of the usual ways to make hydrogel particles. These approaches exhibit restricted size control and notable polydispersity [35]. The production of hydrogel particles via microfluidic methods offers considerable benefits compared with conventional procedures. One significant benefit is that particle size and shape can be carefully controlled. This makes it easier to change properties like flow and viscosity in microfluidic devices so that the results are always the same [3,60].

Furthermore, these technologies provide exceptional reproducibility by enabling the maintenance of consistent conditions throughout the synthesis process. Although microfluidics operates on a small scale, parallel platforms enable scalable production [58]. Microfluidics is a more sustainable method, as it facilitates waste reduction by decreasing reagent consumption and waste production. A significant benefit is the ability to engineer particles that react to stimuli, such as pH or temperature, thereby augmenting their utility in biomedical applications. Because of these benefits, microfluidics is a useful and effective tool for making hydrogel particles in many scientific fields.

Table 2 consolidates research on the synthesis of hydrogel particles utilizing microfluidic devices. The advancements in microfluidic device design and their capacity to tailor materials and techniques for biomedical applications are evident. Microfluidic devices have made it easier to make several systems that release active ingredients that are trapped in polymeric matrices, mostly in spherical shapes. Devices synthesize micro- and nanohydrogels within their microchannels, following the principles of emulsion or droplet formation across distinct phases.

## 9. Conclusions and Future Directions on the Application of Microfluidics in the Production of Nanomedicines

The properties of hydrogels in biological applications meet diverse criteria and obstacles in healthcare and pharmaceuticals. A variety of polymeric materials can be utilized individually or in combination for biological purposes. These materials also satisfy the criteria for application in biological and clinical investigations. Microfluidics has transformed the production of nanoparticles and systems for controlled drug delivery. This approach offers considerable benefits in the production of nanomedicines with very specialized characteristics. The adaptability of microfluidic devices has facilitated the fabrication of hydrogels and other polymeric materials for biological applications, including tissue regeneration and targeted drug delivery.

Microfluidics offers a viable solution for the construction of numerous systems, presenting substantial benefits through the individualization of devices adapted for specific systems and requirements, including diverse materials, carriers, active principles, microorganisms, dimensions, and configurations. This approach facilitates the regulation of manufacturing and operational processes, resulting in more uniform pharmacological effects of nanomedicines. From the perspective of the pharmaceutical industry, it is an invaluable instrument that facilitates continuous operations and enables devices to collaborate, resulting in enhanced and profitable yields. Microfluidics offers a feasible approach for advancing diverse systems, providing multiple benefits.

In combination, microfluidics may open a field for integrating biopolymers with different pharmaceutical forms that would be biocompatible with the host, as well as favor a controlled release for extended effects of drugs. Also, with the use of biopolymers, environmentally friendly products may contribute to reducing contamination due to the acquisition of common polymers. In other applications, encapsulation of bioactive compounds or probiotics through microfluidics could be introduced in food formulations to produce functional foods.

The utilization of diverse materials such as transporters, active chemicals, microorganisms, dimensions, forms, and additional components facilitates the creation of devices tailored to individual systems and requirements. This approach facilitates the regulation of manufacturing and operational processes, resulting in more uniform pharmacological effects of nanomedicines. From the pharmaceutical industry’s perspective, it is an invaluable instrument that enables continuous operations and the integration of devices to achieve enhanced yet profitable yields. In the future, the integration of microfluidics with emerging technologies such as 3D bioprinting and nanotechnology may facilitate the development of more effective and accurate pharmaceuticals, enhancing their influence on contemporary medicine.

## Figures and Tables

**Table 1:** Overview of several devices employed for particle generation in biomedical applications. HFF—hydrodynamic flow focusing. SHM—staggered herringbone mixer.

Year	Device	Material of Device	Device Manufacturing Technology	Author/References
2004	2D HFF	Silicon/glass; PDMS	Photolithography on silicon	[65]
2008	2D HFF	Silicon/glass; PDMS	Soft lithography on PDMS (SU-8)	[121]
2009	SHM	PDMS; Cyclicolefin copolymer (COC)	Floating coating drug delivery system	[122]
2010	2D HFF	Silicon/glass; PDMS	Photolithography and deep reactive ion etching (DRIE) on silicon	[123]
2011	3D HFF	Glass capillaries; PDMS	Soft lithography on PDMS	[124]
2012	2D HFF, T-junction, flow focusing, and co-flowing	Silicon/glass; PDMS	Photolithography process on glass	[125]
2012	3D HFF variants	PDMS; glass capillaries	Photo- and soft lithography on PDMS (SU-8)	[61]
2012	Droplet mixer	PDMS; glass capillaries	Capillary droplet reactor and silicone tubing	[126]
2012	Droplet mixer	PDMS; glass capillaries	Photo- and soft lithography on PDMS (SU-8)	[127]
2012	Jet mixers (MIVM	Polycarbonate and PTFE tubing; Teflon tubing	Silicon/Pyrex microfluidic device	[128]
2013	2D HFF	Silicon/glass; PDMS	Flow-focusing application camera	[40]
2013	3D HFF variants	PDMS; glass capillaries	Photo- and soft lithography on PDMS (SU-8)	[129]
2013	3D HFF variants	PDMS; glass	Photo- and soft lithography on PDMS (SU-8)	[130]
2013	Jet mixers (MIVM and CIJ)	Polycarbonate and PTFE tubing; Teflon tubing	Multi-inlet vortex and confined impact jet mixer	[131]
2014	3D HFF	Glass capillaries;	Multi-capillary glass matrix design	[132]
2014	3D HFF	PDMS; glass	Photo- and soft lithography on PDMS (SU-8)	[133]
2014	Jet mixers (MIVM and CIJ)	Polycarbonate and PTFE tubing; Teflon tubing	Coaxial turbulent jet mixer with clear polycarbonate tubes and clear probe	[42]
2015	3D HFF variants	PDMS; glass capillaries	Borosilicate capillary assembly (glass)	[42]
2015	Baffle mixer	PDMS; glass	3D glass capillary device	[42]
2015	SHM	PDMS; Cyclicolefin copolymer (COC)	Microfluidics chip device	[4]
2016	2D HFF	Silicon/glass; PDMS	Photo- and soft lithography on PDMS (SU-8)	[45]
2016	SHM	PDMS; Cyclicolefin copolymer (COC)	Multi-inlet vortex and confined impact jet mixer	[134]
2016	SHM	PDMS; Cyclicolefin copolymer (COC)	Microfluidic mixing system.	[49]
2017	2D HFF	Silicon/glass; PDMS	Soft lithography on PDMS (SU-8)	[46]
2017	2D HFF	Silicon/glass; PDMS	Soft lithography on PDMS (SU-8)	[135]
2017	3D HFF variants	PDMS; glass capillaries	Photo- and soft lithography on PDMS (SU-8)	[136]
2018	2D HFF	Silicon/glass; PDMS	Injection molded propylene	[137]
2018	2D HFF/cross-slot microfluidic	Silicon/glass; PDMS	Soft lithography on PDMS (SU-8)	[138]
2018	Droplet mixer	PDMS; Glass capillaries	Photolithography on PDMS (SU-8)	[48]
2018	Jet mixers (MIVM and CIJ)	Polycarbonate and PTFE tubing; Teflon tubing	Photo- and soft lithography on PDMS (SU-8)	[139]
bezel 2019	3D HFF variants	Glass capillaries	Insertion of conical cylindrical capillaries in a square capillary	[5]
2019	Droplet mixer	PDMS; glass capillaries	Soft lithography on PDMS (SU-8)	[140]

**Table 2:** Overview of different research on the production of polymeric nanoparticles with microfluidic devices. Depending on the structure of the device, the HFF—hydrodynamic flow focusing—is usually divided into 2D, focused on the horizontal direction, and 3D, focused horizontally and vertically in tiny fluids. SHM—staggered herringbone mixer.

Year	Device Geometry	Polymer	Drugs	Applications	Author, Year
2008	2D HFF	PLGA-PEG	---	Drug delivery	[121]
2010	2D HFF	PLGA-PEG	Lecithin	Sustained release drug delivery	[47]
2011	3D HFF	PLGA-PEG	---	Drug delivery	[124]
2012	2D HFF, T-junction, flow focusing, and co-flowing	Hyaluronic acid (HA)	---	Drug delivery and cosmetic field	[125]
2012	3D HFF variants	PLGA + LIPIDS + PEG	---	Controlled release	[61]
2013	2D HFF	PLGA	Gemcitabine	Drug delivery in cancer	[40]
2013	3D HFF variants	PLGA-PEG	Docetaxel	Prostate cancer	[129]
2014	2D HFF	PLGA-PEG	---	Drug delivery	[142]
2014	3D HFF	PLGA-PEG	Docetaxel	Drug delivery	[133]
2015	SHM	LNPs: phospholipids, cholesterol and polyethylene glycol (PEG)	---	RNA delivery	[4]
2016	SHM	PCL-b-PEG in THF.	Paclitaxel-VES combined/VES	Enhanced nanoparticle formation	[134]
2016	SHM	polyethylene glycol lipid (PEG)	Ionizable amino-lipid, diaryl-noleoylme-thyl-4-dimethylaminobutyrate (DLin-MC3-DMA)	LNP-siRNA for RNA delivery	[49]
2017	2D HFF	DOPE, DOTAP, DOPC and DSPE-PEG (2000)	mNALPs with folate-conjugated	Gene tumor targeting	[46]
2017	3D HFF variants	Pobi(beta-amino ester) PBAE	Plasmid DN	Gene delivery for gene therapy	[136]
2018	2D HFF/cross-slot microfluidic	PLGA	Curcumin	Drug delivery	[143]
2018	Droplet mixer	lipid/alcohol	Interfering RNA (siRNA) DNA or RNA/buffer	Nanomedicine drug delivery systems	[48]
2018	2D HFF	PLGA	Model protein (OVA)	Vaccine adjuvants	[144]

## Data Availability

No new data were created.
